# Electrocardiogram-derived respiratory rate: State-of-the-art and implications for remote cardiopulmonary monitoring

**DOI:** 10.1038/s41746-026-02720-4

**Published:** 2026-05-13

**Authors:** Carmen Martínez Antón, Zakaria El Ghebouli, Vladimír Sobota, Amaël Mombereau, Laura Bear, Jesse D. Roberts, Kanchan Kulkarni

**Affiliations:** 1 University of Bordeaux, INP, IMB, UMR 5251, IHU Liryc, F-33000 Bordeaux, France; 2 University of Bordeaux, INSERM, CRCTB, U1045, IHU Liryc, F-33000 Bordeaux, France; 3 Cardiovascular Research Center, Department of Medicine, Massachusetts General Brigham, Boston, MA USA

**Keywords:** Cardiology, Computational biology and bioinformatics, Diseases, Engineering, Health care, Medical research

## Abstract

Accurate respiratory rate (RR) measurement is essential for early detection of physiological deterioration, yet conventional approaches remain limited by poor robustness and restricted suitability for continuous or remote monitoring. ECG-derived respiratory rate (EDRR) offers a non-invasive alternative by exploiting respiration-induced modulations in cardiac electrical activity, including morphological changes, heart rate variability, and their combined effects. This review provides a structured synthesis of EDRR methods, spanning physiological mechanisms, signal acquisition and preprocessing considerations, and algorithmic approaches including morphology-based, autonomic (heart rate variability/spectral), and fusion strategies. We further evaluate datasets, validation practices, and performance trade-offs across controlled and real-world conditions. Key challenges include susceptibility to motion artifacts, inter-subject variability, inconsistent evaluation protocols, and limited generalizability in ambulatory settings. Addressing these limitations is critical for translating EDRR techniques into robust, scalable solutions for wearable and telehealth-based respiratory monitoring.

## Introduction

Respiratory rate (RR) is a fundamental vital sign that provides critical insight into pulmonary and systemic physiological status^1^. Accurate RR assessment is particularly important in emergency and critical care settings, as well as in the longitudinal management of chronic respiratory disease^2^. Deviations from baseline RR may signal early physiological deterioration, exacerbation of asthma or chronic obstructive pulmonary disease, progression of pneumonia, or impending respiratory failure^3^. Timely recognition of such changes can reduce morbidity and prevent emergent hospital admission.

Abnormal RR patterns are also associated with adverse cardiovascular outcomes^4^. Sleep-disordered breathing (SDB), including obstructive sleep apnea (OSA), is associated with increased risk of heart failure and coronary artery disease^5,6^. Although often considered a condition of adulthood, OSA affects an estimated 1–5% of children globally, with a large proportion remaining undiagnosed^7^. In pediatric populations, SDB is additionally associated with neurodevelopmental impairment and adverse cardiopulmonary outcomes. These observations underscore the need for reliable, non-invasive, and scalable RR monitoring technologies across diverse age groups and clinical contexts.

Recent advances in telehealth and wearable technologies have enabled continuous remote monitoring of cardiopulmonary signals^8^. Lightweight, battery-powered devices improve patient adherence and allow longitudinal assessment outside of hospital settings^9–11^. However, conventional RR measurement methods remain limited in portability and robustness. Respiratory inductive plethysmography, although considered a reference standard, requires thoracic and abdominal bands that may be uncomfortable and susceptible to positional artifacts. Capnography and airflow-based measurements require dedicated hardware and may be unreliable in ambulatory conditions^2,8^. Manual breath counting is observer-dependent and prone to variability. Collectively, these limitations constrain widespread deployment of continuous respiratory monitoring.

Electrocardiogram-derived respiratory rate (EDRR) offers an alternative approach by leveraging respiration-induced modulations of the cardiac electrical signal. Changes in thoracic impedance, cardiac axis orientation, waveform amplitude, and beat-to-beat interval variability provide physiologically grounded surrogates of respiration. Because ECG signals are already routinely acquired in clinical care and increasingly available in wearable platforms, EDRR enables RR estimation without additional dedicated respiratory sensors. This dual use of ECG data supports integrated cardiopulmonary monitoring while reducing sensing burden.

Although numerous EDRR algorithms have been proposed and validated under controlled laboratory conditions, clinical translation has lagged. Reported performance varies substantially depending on signal quality, motion artifacts, lead configuration, physiological variability, and population characteristics. At the same time, the rapid expansion of wearable ECG technologies has created new deployment contexts in which signal quality, computational efficiency, and robustness to motion are critical. As a result, methods that perform well in curated datasets may not generalize to ambulatory or heterogeneous real-world environments.

Compounding this challenge, the EDRR literature has grown in a largely method-centric and heterogeneous manner, with inconsistent validation protocols, performance metrics, and population representation. This has made systematic comparison difficult and has obscured the conceptual relationships among morphology-based, heart rate variability (HRV)-based, and fusion approaches. Several prior reviews have summarized EDRR methodologies and comparative performance across datasets and signal-processing strategies^12,13^. While these contributions provided important benchmarking perspectives, the continued expansion of wearable technologies, signal-fusion strategies, and translational deployment frameworks warrants renewed synthesis. A synthesis that clarifies methodological taxonomy, highlights performance trade-offs, and frames EDRR within translational constraints is therefore timely.

In this Review, we organize the literature around the key components of EDRR systems: signal acquisition and preprocessing, respiratory rate estimation, and technology deployment considerations. Within this structure, the respiratory estimation methods are categorized into three principal groups: (1) beat morphology-based approaches that exploit respiration-induced waveform modulation, (2) HRV and spectral approaches that leverage respiratory sinus arrhythmia (RSA), and (3) fusion strategies that combine complementary signal features to enhance robustness.

Our synthesis extends beyond estimation algorithms to include reviewing upstream and downstream factors that shape the performance and applicability of EDRR. We examine how ECG configuration and preprocessing constrain extractable respiratory information and influence method selection. In addition, we review validation, hardware, and deployment considerations that affect the real-world performance of EDRR. By adopting this end-to-end perspective, we aim to provide a comprehensive account of the state-of-the-art that links signal acquisition, algorithmic design, and translational implementation, supporting the progression of EDRR toward clinically robust and scalable cardiopulmonary monitoring.

## Physiological basis of ECG-derived respiratory rate

**Fig. 1:**
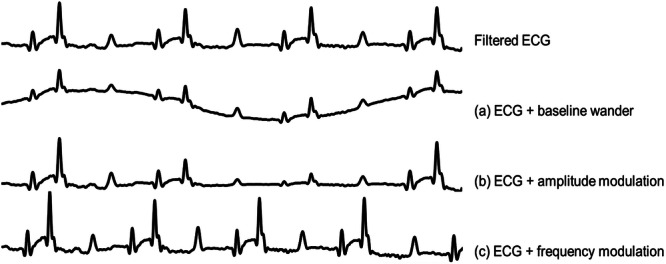
Respiratory effects on an exemplary ECG signal. **a** ECG with baseline wander caused by chest movement, **b** ECG with amplitude modulation due to variations in electrical axis rotation, and **c** ECG with frequency modulation or respiratory sinus arrhythmia (RSA), where heart rate fluctuates with respiration.

EDRR is grounded in well-established cardiopulmonary coupling mechanisms (Fig. 1)^12,14^. Respiration modulates the ECG through mechanical displacement of the heart within the thorax and through autonomic regulation of cardiac rhythm. These interactions produce reproducible baseline, amplitude, and frequency modulations in the ECG that can be leveraged for RR estimation.

Respiratory motion induces baseline wander as diaphragmatic excursion and thoracic volume changes alter the heart’s position relative to surface electrodes (Fig. 1a)^15^. Concurrently, inspiratory and expiratory shifts in cardiac orientation modify QRS morphology and R-wave amplitude due to rotation of the electrical axis and changes in thoracic impedance (Fig. 1b)^16^. During deep inspiration, caudal-cranial translation of up to 4.9 mm and rotational shifts of approximately 1.5° have been reported^17^, illustrating the mechanical basis for amplitude modulation. These morphology-based modulations provide the physiological substrate for beat-to-beat amplitude and vector-based EDRR approaches^17,18^.

Respiration also influences ECG frequency through RSA, a manifestation of autonomic modulation^19^. Inspiration transiently suppresses vagal tone, increasing heart rate, whereas expiration restores vagal influence and slows the heart^20^. This cyclical variability contributes to HRV and enables frequency-domain estimation of RR from R-peak interval fluctuations (Fig. 1c). However, the magnitude and stability of RSA depend on age, autonomic integrity, fitness level, medication exposure, and pathological conditions^18,21,22^. For example, preterm infants exhibit irregular or intermittent RSA reflecting immature autonomic control, underscoring developmental considerations relevant to neonatal monitoring^21^.

Importantly, the reliability of EDRR is influenced by physiological and anatomical context. Breathing depth, body position, pregnancy, and intra-abdominal conditions alter cardiac orientation and modulation strength^17^. Cardiopulmonary diseases,including chronic obstructive pulmonary disease, pulmonary embolism, ventricular hypertrophy, and conduction abnormalities,can further modify the respiratory signature embedded in the ECG^22,23^. In arrhythmias such as atrial fibrillation, respiratory modulation of beat-to-beat intervals becomes less reliable, limiting HRV-based estimation strategies^24^. These physiological and anatomical factors underpin the interpretation and selection of EDRR methodologies.

## Search strategy

Google Scholar and PubMed were searched without language or time restrictions using the terms (“ECG” OR “electrocardiogram”) AND (“respiration” OR “respiratory rate” OR “breathing” OR “breath rate”). Studies were included if they described EDRR estimation methods evaluated in preclinical or clinical settings, with ECG serving as the primary signal source.

The search prioritized representative methodological approaches, validation strategies, and performance benchmarks that reflect the current state-of-the-art and its translational potential. Studies focused on non-ECG modalities or lacking validation for clinical or wearable deployment were excluded.

## ECG-derived respiratory rate estimation

Since its introduction in 1985^25^, EDRR has evolved from proof-of-concept signal extraction into a diverse methodological ecosystem shaped by clinical need, hardware constraints, and deployment context. Rather than progressing linearly, EDRR development reflects three dominant conceptual paradigms: (1) beat morphology–based approaches that exploit respiration-induced modulation of ECG waveform features; (2) HRV–based approaches that leverage autonomic coupling through respiratory sinus arrhythmia; and (3) fusion strategies that integrate multiple respiratory surrogates to improve robustness.

**Fig. 2:**
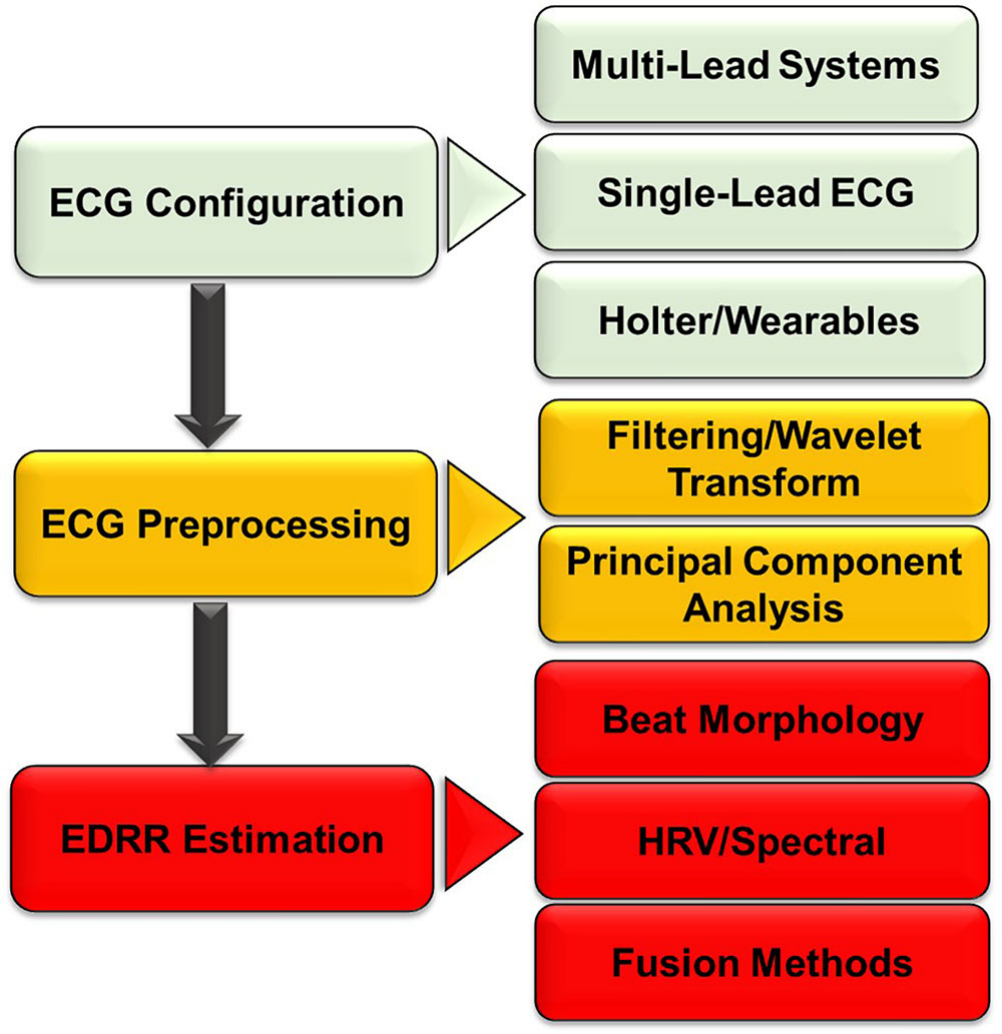
Conceptual overview of ECG-derived respiratory rate (EDRR) methodologies. The pipeline highlights three key stages: ECG configuration, preprocessing techniques, and respiratory rate estimation approaches, including beat morphology-based, HRV/spectral, and fusion methods.

This taxonomy provides a framework for comparing methodological assumptions, computational complexity, validation rigor, and real-world applicability. In parallel, advances in ECG hardware from multi-lead clinical systems to wearable single-lead devices have reshaped algorithm design priorities, shifting emphasis toward signal robustness, computational efficiency, and deployment feasibility. Figure 2 depicts an overview of the key components involved in EDRR estimation. Importantly, ECG configuration and preprocessing are discussed here not as generic signal-processing topics, but as boundary conditions that directly constrain which respiratory modulations can be observed and which estimation paradigms (morphology-based, HRV/spectral, or fusion) are feasible in a given deployment context.

### ECG configurations

ECG configuration is a primary determinant of EDRR feasibility, performance, and deployment context. Lead number and spatial orientation directly influence which respiration-induced modulations—baseline wander, amplitude changes, axis rotation, or interval variability—are observable and how robustly they can be extracted. Because no universal electrode placement optimally captures respiratory effects across all populations, EDRR performance depends on both the hardware configuration and the individual’s anatomy^18^. Physiological and anatomical factors, including body habitus, posture, and underlying cardiopulmonary conditions, therefore indirectly shape the effectiveness of a given ECG configuration by modulating the strength and visibility of respiratory signatures.

Early EDRR methods predominantly relied on multi-lead clinical systems under assumptions of approximate lead orthogonality, enabling vectorcardiographic representations and separation of spatial respiratory effects. While these configurations provided rich spatial information and improved feature stability, they were inherently tied to clinical-grade hardware and controlled acquisition environments.

In contrast, contemporary EDRR development increasingly targets systems with a reduced number of leads and single-lead configurations, driven by the rapid adoption of wearable and ambulatory monitoring platforms. These systems prioritize minimal hardware complexity, low power consumption, and patient comfort, but sacrifice spatial redundancy. As a result, algorithm design has shifted toward lead-agnostic approaches, adaptive feature extraction, signal quality indexing, and computational efficiency.

Hardware evolution from stationary multi-channel systems to compact wearable patches and chest-worn devices has further reshaped signal characteristics. Reduced spatial sampling, non-orthogonal electrode placement, motion-induced artifacts, and variable contact impedance alter the observability and stability of respiratory modulation. Consequently, modern EDRR algorithms must explicitly account for acquisition-dependent constraints, emphasizing robustness, adaptive processing, and validation across heterogeneous hardware environments.

Collectively, ECG configuration is not merely a hardware detail but a defining constraint that shapes feature selection, computational strategy, and translational scalability. For example, morphology-based approaches are particularly sensitive to spatial orientation and amplitude stability, whereas HRV-based methods depend primarily on reliable R-peak detection and are therefore less dependent on multi-lead spatial redundancy. Fusion strategies may partially compensate for reduced lead information by integrating interval- and morphology-derived features, but remain constrained by acquisition quality.

### ECG preprocessing

Preprocessing is essential to isolate respiration-induced modulations from baseline drift, high-frequency noise, motion artifacts, and powerline interference. Because EDRR relies on subtle amplitude, baseline, or interval fluctuations embedded within the ECG, signal conditioning directly affects respiratory observability and downstream estimation accuracy.

Baseline wander, often caused by electrode motion or respiratory chest displacement, is commonly addressed through high-pass filtering^26,27^ or adaptive detrending^14^. Band-pass filtering^28^ is frequently applied to preserve QRS morphology while attenuating low-frequency drift and high-frequency muscle artifacts. The choice of filter design—finite impulse response^26^ versus infinite impulse response, linear-phase versus recursive structures—reflects trade-offs between phase preservation, computational efficiency, and real-time deployment requirements^29^.

Powerline interference (50–60 Hz) and electromyographic noise are typically suppressed through notch or band-limited filtering^26^. Importantly, respiration-induced modulations occur at substantially lower frequencies (approximately 0.1–0.5 Hz), allowing temporal separation from electrical and muscular contaminants. This separation of timescales enables envelope extraction and frequency-domain analysis that are relatively robust to high-frequency disturbances^30^.

Sampling frequency also influences EDRR reliability. Although respiratory oscillations occur at low frequencies, accurate R-wave detection and morphology tracking require sufficient temporal resolution. Clinical systems commonly sample at 250–500 Hz to preserve waveform fidelity^31^, while lower sampling rates may be acceptable for interval-based analysis when spectral precision is not required. In wearable contexts, sampling decisions must balance power constraints with the need for reliable fiducial detection and artifact mitigation.

Collectively, preprocessing is not merely a denoising step but a determinant of respiratory signal integrity. Filter selection, sampling strategy, and artifact handling shape which respiratory modulations remain accessible and ultimately influence algorithm robustness across clinical and ambulatory environments. The relative importance of specific preprocessing steps varies by estimation paradigm: morphology-based methods require phase-preserving filtering to avoid distortion of amplitude and axis features, HRV-based approaches prioritize precise R-wave detection and interval stability, and fusion strategies depend on consistent feature extraction across both domains.

These configuration and preprocessing considerations directly shape the extraction of respiratory information from ECG signals. Depending on which signal components are preserved and how fiducial points are detected, respiratory rate can be inferred through distinct analytical paradigms. These approaches can be broadly categorized into morphology-based, HRV/spectral, and fusion strategies.

### EDRR estimation methods

At present, no formal regulatory or engineering standard defines accuracy thresholds, validation protocols, or reporting conventions for EDRR algorithms. Reported performance, therefore, varies widely across studies, reflecting differences in datasets, hardware configuration, and evaluation methodology rather than intrinsic algorithmic superiority. Contemporary EDRR approaches commonly report mean absolute errors in the range of 2–6 breaths per minute (bpm)^32^ under controlled conditions, with relative errors of approximately 5–10% in selected cohorts^33–35^.

Although a frequently cited clinical benchmark is agreement within ±2 bpm of a reference standard, absolute error alone can be misleading across age groups and baseline respiratory rates. For example, a 2 bpm deviation represents a 14% relative error at a baseline RR of 14 bpm (typical adult resting respiration) but only 5% at 40 bpm (typical newborn respiration^36^). Consequently, comprehensive evaluation requires multiple complementary metrics, including absolute and relative error, agreement analyses, correlation coefficients, and coverage or availability measures.

The heterogeneity of performance reporting has complicated direct comparison across platforms and study populations. To support structured interpretation, Table 1 summarizes commonly reported evaluation metrics and their clinical context, and Table 2 provides a consolidated overview of published EDRR methodologies, hardware configurations, and validation settings.

**Table 1:** List of commonly used performance evaluation metrics and statistical methods

Metric	Definition	Typical clinically acceptable values	Clinical interpretation	Key citations
Mean absolute error (MAE)	Mean of the absolute difference between the estimated RR and the reference RR	≤1 bpm (excellent); 1–2 bpm (good); >3 bpm (poor)	Directly interpretable RR error; most commonly reported metric	Charlton et al.^12^; Li et al.^78^
Root mean square error (RMSE)	Square root of the mean squared RR error; penalizes large deviations	≤2–3 bpm acceptable; >4 bpm poor	Sensitive to outliers and missed breaths	Roberts et al.^2^; Nemati et al.^55^
Relative/percentage error (RE)	Absolute RR error normalized by reference RR (%)	<5% excellent; 5–10% acceptable; >15% poor	Useful across wide RR ranges (exercise, pediatrics)	Sobron et al.^46^; Sarkar et al.^60^
Pearson correlation/correlation coefficient (*r*)	Linear correlation between the estimated and reference RR time series	≥ 0.9 strong; 0.7–0.9 moderate	Captures trend tracking but not bias	Charlton et al.^12^; Roberts et al.^2^
Concordance correlation coefficient (CC)	Measures correlation + agreement (bias and scale)	≥0.9 excellent; ≥0.75 acceptable	Preferred over Pearson Correlation for interchangeability	Cysarz et al.^50^
Bland–Altman bias & limits of agreement (LoA)	Mean RR difference ± 1.96 SD	Bias ≈0 bpm; LoA within ±2–3 bpm	Gold standard for clinical agreement	Charlton et al.^12^; ISO 80601-2-61
Spectral/magnitude-squared coherence	Similarity of frequency content between EDRR and reference respiration	≥0.8 strong; 0.6–0.8 moderate	Validates waveform-level respiratory extraction	Bailón et al.^54^
Sensitivity/PPV (event detection)	Correct detection of RR or apneas, hypopneas, and abnormal RR events	≥90% strong; 80–90% acceptable	Relevant for sleep-disordered breathing	Koff et al.^10^
Coverage/availability (%)	Percentage of time RR is successfully estimated	≥90% robust; <70% poor	Critical for continuous and wearable monitoring	Widjaja et al.^41^; Liu et al.^79^
SQI-weighted error metrics	Errors computed only on high-quality segments	Lower error with high coverage preferred	Balances accuracy vs reliability	Orphanidou et al.^80^

**Table 2:** Chronological list of published studies of EDRR algorithms, highlighting algorithmic approaches, performance metrics, and study characteristics

Year	Author and reference	Method of estimation (ECG features used)	Performance (if quantitative)	ECG configuration (no. of leads used)	Study type (clinical/animal/simulation)
1985	Moody^25^	Mean electrical axis deviation	NR	2-lead	Clinical (MIT-IBH + AHA database)
1992	Khaled^28^	R-wave peak modulation	NR	12-lead	Clinical (healthy pediatric)
1994	Zhao^52^	Spectral central frequency	$\mathrm{CC}$ = 0.9977	2-lead	Clinical (healthy subjects)
2001	Mason^37^	Changes in QRS amplitude	$\mathrm{Sen}$ = 0.7689	Single-lead	Clinical (MIT-IBH + AHA database)
2003	Mazzanti^44^	Changes in QRS amplitude	$\mathrm{Sen}$ = 0.98	12-lead	Clinical (OSA patients)
2006	Bailón^54^	Electrical axis rotation angle variations	$\bar{\varepsilon }$ = 5.9% ± 4%	12-lead	Clinical + simulated (stress test)
2007	O’Brien^49^	Changes in QRS + demodulation	$\mathrm{CC}$ = 0.75; $\mathrm{Acc}$ = 0.82	Single-lead	Clinical (Apnea-ECG database)
2008	Cysarz^50^	R-wave peak modulation	${\rho }_{c}$ = 0.74	Single-lead	Clinical (healthy subjects)
2008	Bowers^48^	Principal component analysis	$\mathrm{MAE}$ ≤ 0.2 bpm	Single-lead	Clinical (healthy subjects)
2009	Arunachalam^29^	R-wave peak modulation	NR	Single-lead	Clinical (Physionet Fantasia database)
2009	Boyle^53^	Band-pass filter + R_peak_-R_peak_ interval	$\mathrm{MAPE}$ $\sim$ 17%	Single-lead	Clinical (healthy subjects)
2009	Pucik^56^	Changes in QRS amplitude	NR	Single-lead	Clinical (healthy subjects)
2010	Dash^57^	Time-frequency spectrum methods (2)	$\widetilde{\varepsilon }$ ≈ 2.5% ± 2.64%	Single-lead	Clinical (healthy subjects)
2010	Langley^47^	Principal component analysis features (4)	$\mathrm{CC}$ = [0.56, 0.97]	Single-lead	Clinical (healthy subjects)
2010	Nemati^55^	Modified Kalman Filter-based ECG	$\bar{\mathrm{RMSE}}$ ≈ 4.21	Single-lead	Clinical (polysomnography data)
2010	Sobron^46^	Comparison of methods (4)	$\widetilde{\varepsilon }$ = [0.7, 42.7] %	15-lead	Clinical (Physionet Fantasia + healthy)
2010	Widjaja^41^	Comparison of methods (4)	$\min (\mathrm{MSE}$) ≤ 0.63	3-lead	Clinical (pregnancy stress test)
2011	Babaeizadeh^42^	Mean electrical axis deviation	$\mathrm{Sen}$ = 0.45; $\mathrm{Acc}$ = 0.61	Single-lead	Clinical (suspected SDB subjects)
2011	Madhav^58^	Empirical Mode Decomposition	$\bar{\mathrm{Acc}}$ = 0.9967	Single-lead	Clinical (Physionet MIMIC database)
2013	Lázaro^45^	QRS slopes	$\min (\varepsilon$) = -1.07% ± 8.86%	17-lead	Clinical (stress test)
2013	Liu^62^	Power spectrum analysis from HRV	$\widetilde{\varepsilon }$ ≈ 15% ± 5%	Single-lead	Clinical (healthy subjects)
2013	Orphanidou^16^	Respiratory poles from fused data	$\bar{\mathrm{MAE}}$ = 0.825; $\widetilde{\varepsilon }$ ≈ 4.85%	Single-lead	Clinical (Physionet Fantasia database)
2014	Helfenbein^19^	Peak-to-trough QRS amplitude	$\mathrm{Sen}$ = 0.99; $\mathrm{PPV}$ = 0.97	Single-lead	Clinical (polysomnography data + MRI)
2014	Lázaro^81^	QRS slopes + R-wave angle	$\varepsilon$ = 0.5% ± 4.1% for tilt test	17-lead	Clinical (stress test + tilt test)
2014	Sinnecker^43^	ECG morphology-derived features (10)	$\mathrm{AUC}$ = 0.74	3-lead	Clinical (myocardial infarction)
2014	Mirmohamadsadeghi^64^	Common frequency tracking	$\mathrm{MAE}$ = [1.07, 1.78]	Single-lead	Clinical (Physionet Fantasia database)
2014	Weiss^77^	SNR root-mean-squared QRS amplitude	$\bar{{R}^{2}}$ = 0.965	12-lead	Animal (ventilated swine)
2015	Sarkar^60^	HRV vs. Peak Amplitude Variation	$\bar{\mathrm{MAE}}$ = ± 0.57 vs. ±0.7 bpm	Single-lead	Clinical (Physionet Fantasia + real-time)
2016	Nayan^72^	RR peak interval vs. time data	$\mathrm{MAE}$ = 0.7; $\mathrm{CC}$ = 0.98	Single-lead	Clinical (Physionet MIMIC-II + healthy)
2016	Birrenkott^66^	Signal quality indexes using frequency	$\triangle \mathrm{MAE}$ = [3 ± 0.12, 4.95 ± 0.11]	Single-lead	Clinical (CapnoBase + MIMIC-II)
2016	Charlton^12^	A combination of previous techniques	$2(\mathrm{SD})$ = [4.7, 5.9]	Single-lead	Simulated + clinical (healthy subjects)
2016	Noriega^40^	Final directions of maximum projection	$\mathrm{MAE}$ = [2.82, 4.52]	3-lead	Clinical (MGH/MF Waveform)
2018	Pambianco^39^	Segmented-Beat Modulation Method	$\Delta$ = [0 ± 0.02, 0.01 ± 0.03]	Single-lead	Clinical (CEBS database)
2018	Nayan^26^	RSA + frequency-induced variation	$\mathrm{MAE}$ = 1.25 vs. 1.05	Single-lead	Clinical (Physionet MIMIC-II vs. healthy)
2019	Jorge^21^	Signal quality index above 0.9 using [23]	$\mathrm{MAE}$ = [7.5, 31.7]	Single-lead	Clinical (premature infants)
2020	Kontaxis^71^	Slope range + f-wave suppression	$\widetilde{\varepsilon }$ = 0.015 ± 0.021 Hz	Single-lead	Clinical + simulated (AF patients)
2020	Leube^75^	QRS complex + RR interval	$\varepsilon$ = [-3.1, -1.5]	Single-lead (wrist)	Clinical (sleep laboratory patients)
2020	Varón^68^	Comparison of methods (10)	$\varepsilon$ = [3 ± 8.3, 4.7 ± 10]	Single-lead	Clinical (several databases)
2020	Khreis^13^	Kalman Filter Fusion	$\mathrm{MAE}$ = [0.2, 2.2]	Single-lead	Clinical (CapnoBase + SHERPAM)
2021	Bao^73^	Comparison of methods (4)	$\bar{\mathrm{MAE}}$ = [1.45 ± 0.2, 3.85 ± 0.3]	Single-lead	Clinical (healthy subjects)
2021	Lázaro^74^	Low-computational-cost [45]	$\max (\widetilde{\varepsilon })$=$1.12 \% \pm 6.46 \%$	3-lead (armband)	Clinical (healthy subjects)
2021	Dong^82^	Comparison of features (10)	${\mathrm{MAE}}_{\mathrm{time}} < {\mathrm{MAE}}_{\mathrm{freq}}$	Single-lead	Clinical (Physionet Fantasia database)
2021	Chung^59^	Ensemble empirical mode decomposition	$\mathrm{MAE}$ = 0.92; $\mathrm{RMSE}$ = 1.32	Single-lead	Clinical (healthy subjects)
2021	Baker^14^	Neural networks	$\mathrm{MAE}$ = [0.638, 0.821]	Single-lead	Clinical (Physionet MIMIC-III database)
2022	Bawua^69^	QRS complex + RR interval	95% LOA = [−5.13, 3.13]	7-lead	Clinical (ICU patients)
2022	Chan^67^	Amplitude modulation features + U-Net	$\mathrm{MAE}$ = [0.55, 1.91]	Single-lead (patch)	Clinical (healthy subjects)
2023	Duan^38^	Area under the QRS complex	$\mathrm{Sen}$ = 0.90; $\mathrm{PPV}$ ≃0.97	Single-lead	Clinical (healthy subjects)
2023	Pan^65^	FFT-based autoencoder + DCT layer	$\mathrm{MSE}$ = 0.15; $\mathrm{MAE}$ = 0.30	Single-lead (wireless)	Clinical (healthy subjects)
2023	Sbrollini^61^	Support vector machine classifier	$F1$ = 86.59% vs. 80.57%	12- and single-lead	Clinical (healthy subjects)
2024	Massaroni^27^	Vector magnitude unit + [36]	$\mathrm{MAE}$ < 0.7; $\mathrm{MAPE}$ < 6.4%	Single-lead (chest)	Clinical (healthy subjects)
2024	McErlean^63^	Sensor fusion approach	$\mathrm{EARR}$ > 97.97 ± 3.46	Single-lead	Clinical (sleep laboratory patients)
2024	Roberts^2,51^	Oscillationlexes	*R*^2^ ≃0.91; $\mathrm{RMSE}$ = 2.2 bpm	Single-lead	Clinical (healthy) + animal (ventilated sheep)

Performance quantification used the following terms (alphabetically): 2(SD), 2-fold standard deviation. *AUC* area under the curve, ${Acc}$ accuracy, $\overline{{Acc}}$ mean accuracy, *CC* Pearson correlation coefficient, *EARR* percentage accuracy of the average respiratory rate compared to reference defined in ref. ^49^, *F*1 harmonic mean of precision and recall (F1 Score), NR not reported, LOAs limits of agreement, *MAE* mean absolute error, $\overline{{MAE}}$ mean mean absolute error, $\triangle {MAE}$ difference between MAE for 100% and 50% data retention, *MAPE* mean absolute percentage error, *MSE* mean squared error, *PPV* positive predicted value, *R*^*2*^ R-squared, $\bar{{R}^{2}}$ mean R-squared, *RMSE* root mean squared error, $\overline{{RMSE}}$ mean root mean squared error, *Sen* sensitivity, Δ signed differences, *ε* error, $\bar{\varepsilon }$ mean error, $\widetilde{\varepsilon }$ median error, *ρ*_*c*_ concordance correlation.

For conceptual clarity, we organize EDRR algorithms into three principal categories:

1. Beat morphology–based approaches, which extract respiration from waveform amplitude, area, or axis modulation.
2. HRV- or spectral-based approaches, which infer respiration from RSA and beat-to-beat interval variability.
3. Fusion strategies, which integrate complementary features across time and frequency domains to enhance robustness.

## EDRR methods based on beat morphology

Beat-morphology–based methods form the foundation of EDRR. These approaches exploit respiration-induced modulation of ECG waveform features—most commonly QRS amplitude, area, and electrical axis orientation—to estimate RR. By leveraging beat-to-beat changes in waveform geometry, morphology-based techniques provide a physiologically grounded mechanism for respiratory signal extraction.

Early work demonstrated that deviations in the mean electrical axis derived from orthogonal leads could detect respiratory events such as apneas and hypopneas^25^. Subsequent amplitude-based methods focused on R-wave and R–S modulation^28,37^, while later studies confirmed the high predictive performance of QRS modulation under controlled conditions^38^. Collectively, these studies established that respiration-induced spatial and amplitude shifts in cardiac electrical activity provide reliable respiratory surrogates.

A key limitation of morphology-based approaches is sensitivity to lead geometry and signal instability. Many early methods assumed approximate orthogonality between leads when computing QRS area or angular features—an assumption frequently violated in real-world settings, introducing systematic error^39^. In addition, QRS morphology may become less reliable in the presence of noise, motion artifacts, or cardiac abnormalities, constraining robustness in ambulatory environments.

To address these limitations, multi-lead and dimensionality-reduction strategies were introduced. Principal component–based orthogonalization reduced RR estimation error and demonstrated that respiratory information may extend beyond the QRS complex, with T-wave–based fiducial markers occasionally providing greater stability^40^. Comparative studies evaluating QRS amplitude, R–S amplitude, and QRS area reported strong performance of amplitude-based features, though artifact susceptibility remained a concern^41^. Artifact rejection, supervised learning, and integration of interval-based features further improved detection of sleep-disordered breathing and prognostic assessment^42,43^.

While expanded multi-lead systems incorporating vectorcardiographic and non-standard leads achieved improved apnea detection and RR accuracy under controlled and stress conditions^44–46^, practical constraints, including wearability, computational burden, and deployment feasibility, limited scalability of high-lead-count approaches.

Consequently, the field has increasingly converged toward optimized configurations with a reduced number of leads, including single-lead systems. Principal component analysis^47^ and amplitude demodulation applied to single-lead ECG have demonstrated strong agreement with reference respiration under controlled conditions^48–50^. Building on these principles, Roberts and Kulkarni developed a computationally efficient single-lead algorithm based on root mean square QRS amplitude modulation, achieving a root mean square error of 2.2 bpm in humans and 0.07 ± 0.07 bpm in mechanically ventilated sheep^51^. These findings support the feasibility of morphology-driven EDRR in both clinical and translational settings.

Main takeaways from beat morphology-based EDRR:

- Respiratory modulation of ECG morphology provides a physiologically grounded signal source. Beat-to-beat variations in QRS amplitude, area, and electrical axis reflect respiration-induced cardiac displacement and impedance changes, enabling RR estimation and event detection.
- Performance depends strongly on lead geometry and signal quality. Orthogonality assumptions, electrode placement, motion artifacts, and cardiac pathology influence waveform stability, motivating dimensionality reduction, artifact rejection, and adaptive processing.
- Clinical translation has driven convergence toward reduced-lead and single-lead solutions. Although multi-lead systems offer richer spatial information, scalable and wearable applications increasingly rely on optimized limited-lead configurations that balance physiological fidelity with deployment feasibility.

## EDRR methods based on HRV or spectral analysis

In contrast to morphology-based approaches, HRV-based EDRR methods exploit RSA and autonomic modulation of cardiac rhythm. Rather than analyzing waveform geometry, these methods derive RR from beat-to-beat interval variability, making them attractive for single-lead implementations and computationally efficient real-time systems, particularly when morphological features are unstable.

Early spectral analyses demonstrated that the dominant respiratory frequency could be extracted from ECG-derived signals using frequency–domain methods^52^. Subsequent work extended these approaches to dynamic conditions through wavelet decomposition and time–frequency analysis, enabling RR estimation during daily activity and sleep^53^. More advanced spectral frameworks incorporated vectorcardiographic transformations, autoregressive modeling, and Lomb periodograms to improve stability under nonstationary conditions, albeit with increased computational complexity^54,55^.

Time-frequency and adaptive modeling strategies were introduced to address short data segments and noisy environments. Refined spectral HRV analysis demonstrated that respiratory activity could be identified through phase shifts and amplitude modulation in HRV signals, despite challenges such as phase uncertainty, absence of depth calibration, and susceptibility to artifacts as posture changes^56^. Kalman filtering and signal quality indexing improved robustness to artifact and temporal variability, reducing estimation error in realistic datasets^55^. Decomposition-based techniques^57^, including empirical mode decomposition and ensemble variants, provided adaptive extraction of respiratory components without predefined basis functions^58,59^. While these methods enhanced flexibility in nonstationary signals, they increased computational demands and introduced sensitivity to mode selection and noise.

Comparative analyses reveal that HRV-based approaches perform well during paced or resting breathing but degrade under spontaneous or motion-rich conditions^46,60^. Simpler band-pass filtering of heart rate series within predefined respiratory frequency bands offers computational efficiency^41,61^, yet performance depends strongly on breathing regularity and intact autonomic modulation. In ambulatory settings, motion-induced R-peak detection errors remain a primary limitation^62^. These observations highlight the context-dependent nature of HRV-based EDRR.

Overall, HRV-based methods provide a complementary respiratory surrogate grounded in autonomic physiology but exhibit greater sensitivity to autonomic variability, posture, arrhythmias, and motion compared with morphology-based approaches.

Main takeaways from HRV/spectral EDRR:

- HRV-based EDRR leverages autonomic coupling rather than waveform morphology. RSA provides a physiologically meaningful but indirect respiratory surrogate, enabling single-lead and computationally efficient implementations in subjects with intact autonomic modulation.
- Advanced spectral and decomposition techniques improve robustness under controlled conditions but increase complexity. Wavelets, autoregressive modeling, Kalman filtering, and empirical mode decomposition enhance stability in nonstationary signals but introduce computational burden and sensitivity to motion and artifact.
- Performance is highly context-dependent and motivates complementary strategies. HRV-based methods perform well during paced or resting conditions but degrade in ambulatory or irregular breathing scenarios, underscoring the need for hybrid or fusion approaches in real-world deployment.

## Fusion EDRR methods

Recognizing that no single respiratory modulation source is universally stable, fusion-based EDRR strategies integrate complementary signals to improve robustness across variable physiological and environmental conditions. These approaches combine morphology-derived features (e.g., QRS amplitude or area modulation) with HRV-derived metrics such as RSA, leveraging the strengths of each while mitigating their individual weaknesses.

Fusion strategies generally operate at three levels: (1) feature-level integration of multiple respiratory surrogates, (2) adaptive weighting guided by signal or respiratory quality indices, and (3) model-based state estimation using filtering or learned representations. Rather than relying on a single dominant modulation, fusion frameworks dynamically determine which signal components are most reliable under a given condition.

High-dimensional feature integration has been demonstrated by combining amplitude modulation, principal component–derived signals, and nonlinear manifold representations into composite respiratory estimates^63^. Phase synchronization and dimensionality reduction techniques enhance stability by aligning oscillatory structures rather than simply averaging time series. Similarly, adaptive weighting approaches^64^ incorporate signal quality indices to prioritize physiologically plausible or temporally stable respiratory components^14,26,41^. These strategies are particularly beneficial in aging populations or motion-rich environments where individual modulation sources degrade^16,50^. These data highlight the strength of combining RSA with robust signal conditioning and quality filtering typically found in morphology-based techniques. Several studies^16,21,62^ have proposed respiratory quality indices based on descriptors such as Fourier spectra, Hjorth parameters, autocorrelation, and autoregression. These metrics can guide the weighting of different modulations or serve as inputs to fusion models.

Data fusion techniques, state-space, and regression-based frameworks further improve resilience in noisy or asynchronous conditions. Kalman filtering and smoothing techniques consistently reduce error compared with single-modality estimation^13,37^. More recently, hybrid architectures incorporating representation learning alongside frequency-domain processing have demonstrated substantial error reduction while maintaining physiological interpretability^65^. These developments reflect a broader shift toward combining classical signal processing with data-driven modeling.

Collectively, fusion approaches address the context-dependence observed in morphology- and HRV-based methods by introducing redundancy and adaptive weighting^66^. This paradigm is particularly well suited to wearable and ambulatory environments, where signal quality fluctuates, and physiological variability is pronounced.

Main takeaways from fusion EDRR:

- Fusion improves robustness by integrating complementary respiratory modulations. Combining morphology-based and HRV-derived signals reduces dependence on any single modulation source and enhances stability across heterogeneous conditions.
- Adaptive weighting and signal quality assessment are central to performance. Signal and respiratory quality indices enable dynamic selection of reliable features, improving resilience to motion, aging-related autonomic decline, and fluctuating ECG quality.
- Fusion frameworks are particularly suited to real-world deployment. Kalman filtering, dimensionality reduction, and hybrid signal-processing architectures are often combined within fusion frameworks to integrate heterogeneous information sources. This approach appears to consistently outperform standalone methods in wearable and low-quality signal environments, supporting scalable clinical translation.

## Data sources and validation

The reliability of EDRR algorithms depends not only on signal processing strategy but also on the quality and representativeness of validation data. Across the literature, validation has relied on two primary elements: (1) gold-standard respiratory reference signals and (2) publicly available ECG repositories. Variability in either domain substantially influences reported performance and generalizability.

Reference respiratory signals are typically obtained from airflow sensors, respiratory inductance plethysmography, nasal pressure monitors, or impedance pneumography, methods widely regarded as clinical standards for RR measurement^13,47,49,53,56,67^. Additional modalities, including acoustic sensors, diaphragm electromyography, thermal flow meters, and infrared motion tracking, have also been used in specific contexts^21,38,49,68–70^. While these technologies provide high-fidelity respiratory measurements, their integration into wearable or ambulatory settings remains more complex than ECG acquisition, which is already widely embedded in clinical and consumer devices. This asymmetry underlies the appeal of EDRR as a hardware-efficient surrogate.

Public ECG repositories, including MIT-BIH, AHA, and polysomnographic databases, have played a central role in algorithm benchmarking. However, these datasets vary widely in sampling frequency, subject demographics, rhythm characteristics, recording environments, and annotation standards. Performance metrics, including correlation coefficients, sensitivity and specificity, mean absolute error, and root mean squared error, are inconsistently reported across studies, complicating direct comparison and synthesis. A critical limitation of early validation studies was restricted population diversity. Initial algorithms were largely tested in adults with stable respiratory rates^25^. Subsequent work expanded to pediatric and sleep-disordered populations, where respiratory ranges and variability are broader^21,59^. Nonetheless, systematic validation across age extremes and heterogeneous physiological conditions remains limited.

Cardiac rhythm abnormalities represent another important gap. Most EDRR methods assume a sinus rhythm, wherein respiratory modulation of interval and morphology features is predictable. In arrhythmic conditions such as atrial fibrillation, these relationships deteriorate, reducing the reliability of HRV-based techniques and complicating morphology tracking^71^. Although preprocessing approaches have been proposed to mitigate such interference, comprehensive validation in arrhythmic populations remains sparse.

Collectively, the generalizability of EDRR algorithms is constrained by dataset composition, rhythm assumptions, and environmental control. Broader inclusion of diverse age groups, cardiac rhythms, activity states, and real-world noise conditions is essential to establish clinical robustness. Without such validation breadth, reported performance may overestimate real-world reliability.

## Integration of EDRR in real-world applications and wearables

The translational value of EDRR lies in its ability to extract respiratory information from ECG signals that are already acquired for cardiovascular monitoring. This feature positions EDRR as an attractive solution in settings where minimal additional instrumentation, continuous monitoring, and patient comfort are priorities. However, implementation in real-world and wearable contexts introduces design trade-offs that extend beyond algorithmic performance.

A central deployment decision involves lead configuration. While multi-lead systems offer richer spatial information and may enhance feature stability, wearable platforms typically rely on configurations with a reduced number of leads, often single-lead systems, to minimize hardware complexity and power consumption. Single-lead approaches have demonstrated clinically acceptable accuracy in ambulatory settings when paired with signal quality indexing and artifact mitigation^19,72^. Moreover, respiration-induced modulation varies with electrode placement and cardiac orientation, and in some contexts, single-lead configurations may demonstrate greater robustness than multi-lead vector-based methods^39,73^.

Traditional ECG settings, such as Holter monitors, are cumbersome systems that rely on wet electrodes and wires, limiting comfort and mobility^65^. However, signal quality remains the principal bottleneck in wearable EDRR. Motion artifacts, electrode-skin impedance variability, posture changes, and muscle activity can distort morphology and interval features^27^. Innovations in sensor placement and device architecture, such as sternal patch positioning to reduce motion-induced noise, have shown measurable improvements in ECG fidelity and downstream respiratory estimation^67^. However, trade-offs persist between comfort, wear duration, signal amplitude, and robustness^74^.

EDRR has been evaluated in diverse clinical applications, including sleep monitoring, cardiopulmonary stress testing, chronic respiratory disease management, and ambulatory screening for sleep-disordered breathing. In controlled environments, morphology-based and fusion approaches often demonstrate high sensitivity and specificity. Yet performance variability increases during exercise, irregular breathing, or spontaneous movement, where muscular activity and spectral overlap complicate respiratory extraction^54^. These observations reinforce the need for adaptive algorithms in ambulatory deployment.

Beyond respiratory rate alone, EDRR interacts with broader physiological metrics. Respiratory modulation significantly influences HRV indices, particularly in frequency-domain analyses used for sleep staging and autonomic assessment. Failure to account for respiration may distort HRV interpretation. Conversely, joint analysis of RR, HRV, and related signals such as tidal volume may enhance detection of pathological states, including sleep apnea, heart failure, and metabolic disease. In populations with autonomic dysfunction, such as individuals with diabetes, altered respiratory–cardiac coupling further complicates interpretation.

The COVID-19 pandemic highlighted the need for scalable remote respiratory monitoring. Subtle increases in RR may precede overt symptoms, underscoring the potential value of continuous, ECG-based respiratory tracking in telemedicine and population-level monitoring^67^. While EDRR is not a standalone diagnostic modality, its integration into existing ECG-enabled wearables provides a pragmatic pathway for large-scale deployment.

Collectively, successful real-world implementation of EDRR depends not solely on algorithmic accuracy, but on hardware design, signal quality management, physiological context, and workflow integration.

## EDRR in multimodal bioelectrical frameworks

Although ECG-derived respiration can function independently, integrating ECG with complementary physiological signals can enhance robustness, coverage, and interpretability. Multimodal approaches extend the fusion paradigm by incorporating signals that capture respiratory mechanics through different physical mechanisms, such as motion, impedance, optical perfusion, or bioelectrical activity.

Motion-based sensors, including accelerometers and gyroscopes placed on the chest or diaphragm, directly capture thoracic displacement and have demonstrated high agreement with reference respiratory signals^75^. When combined with ECG-derived respiration, these signals can compensate for intermittent degradation of morphology- or HRV-based features and improve detection of abnormal breathing patterns. Similarly, integrating ECG-derived respiratory estimates into respiratory effort sensing pipelines can enhance data continuity and reduce the proportion of unusable segments during quality assessment^76^.

Optical and mechanical signals provide additional complementary information. Photoplethysmography and seismocardiography capture respiratory influences through vascular and mechanical pathways that may be less sensitive to electrode-skin impedance or sweat accumulation^67^. Morphology-based techniques applied to both intracardiac electrograms and surface ECGs further demonstrate that respiratory modulation is detectable across diverse bioelectrical substrates^77^. These observations underscore that respiratory–cardiac coupling is multimodal by nature.

The principal advantage of multimodal frameworks lies in redundancy. When one signal degrades due to motion, posture change, or environmental noise, complementary modalities may preserve respiratory observability. However, multimodal systems introduce increased hardware complexity, synchronization challenges, and power requirements. Thus, the incremental benefit must be weighed against deployment constraints.

As wearable technologies mature, selective multimodal integration rather than wholesale sensor expansion may represent the most practical strategy. Combining ECG with strategically chosen complementary signals can improve reliability in dynamic environments while maintaining feasibility for long-term monitoring.

## Limitations, challenges, and future perspectives

Despite significant methodological advances, EDRR remains constrained by several fundamental limitations. First, signal quality remains highly variable across deployment environments. Motion artifacts, electrode displacement, posture changes, and physiological variability can distort morphology- and interval-based features, particularly in wearable settings. Robust preprocessing, signal quality indexing, and adaptive feature selection partially mitigate these effects, but no single approach has demonstrated universal reliability across all conditions.

Second, generalizability across diverse populations remains incomplete. Many EDRR algorithms have been developed under assumptions of sinus rhythm and relatively stable respiratory patterns. Performance in pediatric populations, elderly individuals with autonomic dysfunction, and patients with arrhythmias or cardiopulmonary disease requires broader and more systematic validation. Without inclusion of these edge cases, reported accuracy may not reflect real-world clinical diversity.

Third, the inherently low sampling rate of EDRR, typically one estimate per heartbeat, limits temporal resolution and sensitivity to subtle respiratory phenomena, such as hypopneas or irregular breathing transitions. Spectral overlap between cardiac and respiratory components, particularly during dynamic states such as random eye movement (REM) sleep or exercise, further complicates reliable estimation.

Artificial intelligence and machine learning offer potential advantages, but primarily at the level of respiratory pattern analysis rather than basic RR extraction. While classical signal analysis methods are effective for estimating mean respiratory rate, data-driven models may improve the detection of breathing patterns such as apnea or hypopnea, dysregulated respiration, or cardiorespiratory coupling. Importantly, these approaches require accurately curated and physiologically representative training datasets to avoid overfitting and ensure interpretability. Black-box models that obscure physiological mechanisms may limit clinical trust and regulatory acceptance.

Future progress will likely depend on three priorities: (1) standardized validation frameworks across heterogeneous datasets and device platforms, (2) adaptive and fusion-based algorithms optimized for ambulatory deployment, and (3) integration of EDRR within broader cardiorespiratory monitoring ecosystems. In this context, EDRR may serve less as a standalone diagnostic modality and more as a scalable component within multimodal digital health systems.

Ultimately, the clinical value of EDRR will depend not only on algorithmic sophistication but also on reproducibility, interpretability, and performance consistency across real-world conditions.

## Conclusions

Over the past four decades, EDRR estimation has evolved from simple waveform-based heuristics to structured signal-processing and adaptive fusion frameworks. Despite this progress, no single methodological paradigm has proven universally superior. Performance remains contingent on lead configuration, signal quality, physiological context, and deployment environment.

A clear conceptual taxonomy has emerged: morphology-based methods exploit respiration-induced waveform modulation, HRV-based approaches leverage autonomic coupling, and fusion strategies integrate complementary signals to enhance robustness. Each paradigm offers distinct advantages and limitations, and their relative performance depends on population characteristics and real-world constraints.

For EDRR to achieve broader clinical adoption, future efforts must prioritize standardized validation across diverse populations, reproducible benchmarking practices, and algorithmic robustness in ambulatory and wearable contexts. While machine learning may enhance higher-level pattern recognition, such as the detection of apnea or dysregulated breathing, the foundational challenge remains ensuring reliable respiratory signal extraction under heterogeneous conditions.

EDRR is unlikely to function as a standalone diagnostic modality. Rather, its value lies in scalable integration within ECG-enabled digital health systems, where respiratory information can augment cardiovascular monitoring without additional hardware burden. In this role, EDRR represents a pragmatic and potentially powerful component of continuous, multimodal cardiopulmonary assessment in both clinical and remote care settings.

## Data Availability

Summary statistics generated in this study will be made available through the GP2 platform. Access to the individual-level data used in the preparation of this article is coordinated by the corresponding authors and governed by the GP2 Tier 2 data access policy, and may be granted upon approval of the request and execution of a Data Use Agreement by the applicant’s institution. GP2 data are available via the AMP PD platform (https://amp-pd.org). Summary statistics from Nalls et al. were retrieved from the GWAS Catalog (https://www.ebi.ac.uk/gwas/), and summary statistics from Foo et al. were obtained from the supplementary materials of the original publication in JAMA Neurology. Publicly available eQTL summary statistics were used for colocalization analyses. Brain eQTL data were obtained from the metaBrain resource described by de Klein et al., and whole-blood cis-eQTL summary statistics were obtained from the eQTLGen consortium website (cis-eQTLs; full cis-eQTL summary statistics).
